# The Expectancy of Life in Cases of Cancer of the Breast

**Published:** 1900-09-15

**Authors:** 


### THE EXPECTANCY OF LIFE IN CASES OF CANCER OF THE BREAST.

In a clinical lecture recently delivered at University College Hospital Mr. Arthur Barker said that at the present moment the outlook of a woman with cancer of the breast was by no means so desperate as, say, 20 years ago, either in regard to the prospect of prolongation of life by operation, or even of obtaining complete immunity from recurrence. From an analysis of a hundred consecutive cases in which he had excised the breast, he showed that 16 per cent. lived over five years, and 33·7 per cent. over three years. He did not, however, claim that such cases were "cured," since a certain and a not inconsiderable proportion had died most likely from recurrence after an interval of immunity of over three years. Still, only 7 per cent. suffered from local recurrence, the disease when it had returned usually affecting the internal parts, which was a good feature of the modern operations, local recurrence being most depressing and painful, while internal generalisation was not so. Some of the cases, indeed, gave ground for hoping that this disease might really be eradicated by the knife, and it was not, he said, too much to hope that, with still further improvements in early diagnosis and treatment, a far larger proportion of cases might in future be saved. Mr. Barker laid down the following axioms: (1) Excision of the breast to be effectual must be early. (2) The overlying skin, subcutaneous fat, pectoral fascia, axillary fat and glands, with the entire breast, must be widely and deliberately removed. (3) During this dissection all division of carcinomatous tissue should be avoided, and if inevitable freshly sterilised instruments should be used. (4) As little direct handling of the parts as possible should take place. (5) Arrest of all immediate bleeding should be accomplished, preferably by forcipressure, and if not by ligature, and all subsequent oozing by elastic pressure and bandaging. Rest for the wound and the patient should be secured for at least two weeks. (6) All this extensive removal can be accomplished with but little risk to the patient in fairly early cases. (7) When the disease has infected the muscles palliation alone can be expected from surgical interference, and it is questionable whether very extensive operations involving risk from shock ought to be performed with only this end in view. Mr. Barker went on to say that the more localised the primary focus of the carcinoma in the breast appeared to be, the more justified was one in running some risk of shock in doing a wide-reaching operation, because there was in such cases a fair prospect of obtaining complete eradication of the disease thereby. The converse rule appeared also to have much to recommend it—viz., that the more wide-reaching the disease the more clearly should the operator keep mere palliation in view, and by limiting his operation avoid the risk of extreme shock.

